# Transcranial Magnetic Stimulation and Working Memory Training to Address Language Impairments in Aphasia: A Case Study

**DOI:** 10.1155/2021/9164543

**Published:** 2021-11-25

**Authors:** Despina Kranou-Economidou, Maria Kambanaros

**Affiliations:** Department of Rehabilitation Sciences, Cyprus University of Technology, Limassol 3036, Cyprus

## Abstract

**Background:**

Traditionally, people with aphasia (PWA) are treated with impairment-based language therapy to improve receptive and expressive language skills. In addition to language deficits, PWA are often affected by some level of working memory (WM) impairments. Both language and working memory impairments combined have a negative impact on PWA's quality of life. The aim of this study was to investigate whether the application of intermittent theta-burst stimulation (iTBS) combined with computerized WM training will result in near-ransfer effects (i.e., trained WM) and far-transfer effects (i.e., untrained language tasks) and have a positive effect on the quality of life of PWA.

**Methods:**

The participant was a 63-year-old Greek-Cypriot male who presented with mild receptive aphasia and short-term memory difficulties. Treatment was carried out using a multiple baseline (MB) design composed of a pretherapy or baseline testing phase, a therapy phase, and a posttherapy/follow-up phase. The treatment program involved iTBS application to the left dorsolateral prefrontal cortex (DLPFC), an area responsible for WM, for 10 consecutive sessions. The participant received a 3-minute iTBS application followed by 30-minute computer-assisted WM training. Outcome measures included a WM screening test, a standardized aphasia test, a nonverbal intelligence test, story-telling speech samples, a procedural discourse task, and a questionnaire addressing quality of life. These measures were performed three times before the treatment, immediately upon completion of the treatment, and once during follow-up testing at 3 months posttreatment.

**Results:**

We found a beneficial effect of iTBS and WM training on naming, reading, WM, reasoning, narrative, communication efficiency, and quality of life (QoL). *Implications for Rehabilitation.* Noninvasive brain stimulation combined with computerized WM training may be used in aphasia rehabilitation to improve WM and generalize to language improvement.

## 1. Introduction

Transcranial magnetic stimulation (TMS) is a noninvasive technique of brain neuromodulation and neurostimulation [1] which produces a brief electric current in the coil to generate a magnetic field, and in turn, it activates neurons in the vicinity of the coil. Recently, there is a growing interest in the area of working memory (WM) improvement through the use of TMS [2]. WM, the ability to temporarily store and manipulate information in the provision of ongoing tasks, is based on the belief that a dedicated system maintains and stores information in the short term and that this system underlies human thought processes [3]. While a range of cognitive abilities has been associated with WM, including reading comprehension, logical thinking, general intelligence, learning ability, and fluid reasoning [4–8], recent investigations explore how cognitive enhancement can support improvements in other areas (i.e., language) in people with neurological impairments [9, 10].

**Figure 1:**
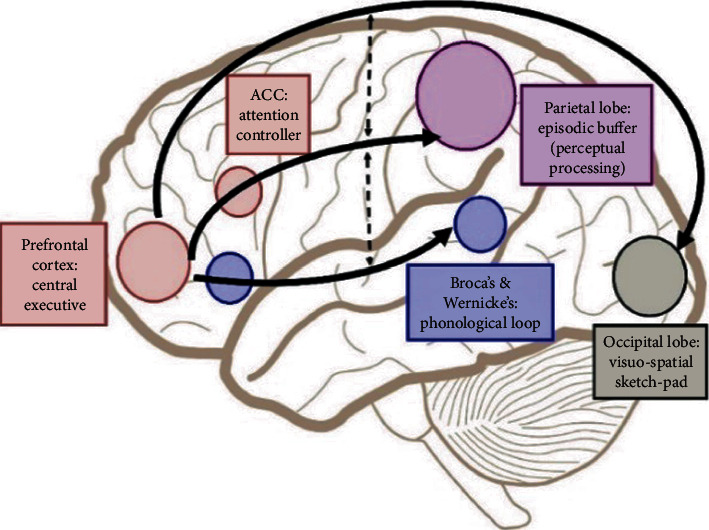
The multicomponent working memory model (Baddeley, 2010) represented simplified as implicated in the brain, in which the central executive assumes the role to exert control and oversee the manipulation of incoming information for intended execution. ACC: anterior cingulate cortex. From Working Memory from the Psychological and Neurosciences Perspectives: A Review by Chai et al., 2018, https://www.ncbi.nlm.nih.gov/pmc/articles/PMC5881171/figure/F1/Copyright© 2018 Chai, Abd Hamid and Abdullah.

Aphasia has traditionally been defined as a language impairment due to the disruption of the blood flow to the brain, which can result in reduced ability to comprehend and/or express oral and/or written language. Lately, research studies investigate whether aphasia is the result of a cognitive process disruption, specifically the disruption of the WM neural network [10]. In the recent past, aphasia was also defined as a cognitive disorder with major linguistic deficits, as opposed to a specific language disturbance [11]. This notion is based on evidence indicating the possible breakdown of an underlying neuronal mechanism that corresponds to a network consisting of several cortices interconnected by white matter tracts [11]. Interestingly, many studies have identified the WM neural network at the frontoparietal area, involving the dorsolateral prefrontal cortex (DLPFC), the anterior cingulate cortex (ACC), and the parietal cortex (PAR) [12–15]. More precisely, the DLPFC has been implicated mainly in tasks demanding executive control such as those requiring integration of information for decision-making, maintenance and manipulation/retrieval of information, and information updating [12]. Chai et al. [16] provided a visual interpretation of Baddeley's theoretical formulation of the multicomponent WM model [17] to specific regions in the human brain as depicted in Figure 1 below. The DLPFC is known for its involvement in WM tasks and for its significant contribution to tasks accuracy [18, 19]. Previous studies have provided evidence of increased activation of the DLPFC during WM tasks [20–22]. In order to achieve a cognitive target such as WM training, WM simultaneously participates in information processing and storage [8]. When WM fails, the ability to carry out many activities of daily living is reduced [23]. Due to this involvement in multiple WM components, the DLPFC is a desirable target for neuromodulation in the context of WM training.

Previous studies have suggested that the neural systems underlying WM capability is plastic, and therefore, WM updating training can lead to WM improvement, particularly in individuals with WM deficiencies [24–26]. Additionally, the effects of training can be transferred to other cognitive functions associated with WM such as general fluid intelligence (Gf) [27]. Gf is defined as the ability to solve novel reasoning problems, and it is associated with comprehension, problem solving, and learning [28]. Gf is a complex ability that enables an individual to adapt their thinking to new cognitive problems and situations [27], and it has been identified to share the same neural networks in the lateral prefrontal and parietal cortices as WM capability [8, 27, 29].

This current study supports that when a stroke occurs, the WM neuronal network is disrupted, resulting in aphasia. The main objective was to explore the potential domains of transfer effect after stimulation of the left DLPFC and WM training and also to measure how efficacious this treatment protocol was for PWA. Specifically, the purpose was to investigate the short-term and long-term combined effects of iTBS and WM training as a mediator to WM improvement, language generalization, and to quality of life (QoL) enhancement. Cognitive (nonverbal), language (verbal), and QoL outcomes are reported at pretherapy (baseline), posttherapy (immediately after the end of the treatment), and follow-up (three months posttreatment).

## 2. Materials and Methods

### 2.1. Inclusion and Exclusion Criteria

The study was carried out at the Rehabilitation Clinic of the Cyprus University of Technology (CUT). Inclusion and exclusion criteria to the study were made available prior to entering the study (Table 1).

The study was approved by the Cyprus National Bioethics Committee, and the participant provided a written consent prior to participating in this study.

### 2.2. Participant Details

**Figure 2:**
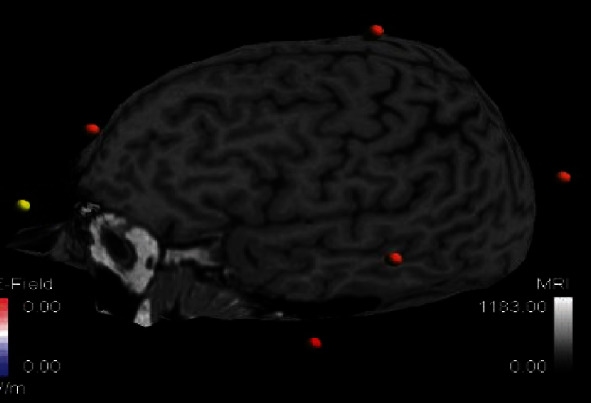
Reported findings of the current MRI noted two small areas of low signal intensity involving the subcortical white matter of the left occipital lobe and the left temporal lobe which were compatible with small areas of brain parenchymal loss.

The participant (C.S.) was a 63-year-old male who suffered a left hemisphere ischemic stroke 45 days prior to the study and was not receiving speech and language therapy. C.S.'s neurologist stated that he experienced mild expressive aphasia with short-term memory (STM) difficulties and verbal information processing difficulties. C.S.'s reported having difficulties remembering recent verbal information while having a conversation with family and friends. He was a retired food and beverage employee, with 12 years of education, and was a hobby farmer. Although brain damage was not visible on the current MRI (Figure 2), the initial medical MRI report indicated the presence of an acute ischemic stroke in the medial temporal lobe. C.S. lived with his wife and did not suffer any paresis or paralysis as he was able to drive and care for himself with minimal assistance.

### 2.3. Data Collection and Procedures

The assessment battery was administered in a predetermined order in 2 sessions, of approximately 2.5 hours duration in total.

#### 2.3.1. Background Tools

The background tools were used to fulfil certain inclusion criteria in order to proceed to the pretesting and treatment stage of this study. A detailed case history was taken including personal and medical information. A TMS safety questionnaire [31] was completed prior to entering the first stage of the inclusion process, followed by a screening procedure which included the following:

- The *Albert's Visual Neglect Test* [32] to determine unilateral spatial neglect
- The *Edinburgh Handedness Inventory* [33] aimed at evaluating handedness of the preferred hand for carrying out daily activities
- The Greek adaptation of the *Beck's Depression Inventory-II* [34, 35], to measure characteristic attitudes and symptoms of depression

#### 2.3.2. Assessment Tools

A battery of tools was administered at baseline, immediately after treatment (same day), and 3 months posttreatment at the follow-up stage. The tools used were as follows:

- The *Raven's Coloured Progressive Matrices* (RCPM) [36]
- Subtests from the Greek version of *Boston Diagnostic Aphasia Evaluation–Short Form* (BDAE-SF) [30]
- The *RehaCom Working Memory Screening Task*
- A *personal stroke narrative* [37]
- The *Multilingual Assessment Instrument for Narratives* (MAIN) [38]
- A *Procedural Discourse* task [39]
- The *Stroke and Aphasia Quality of Life Scale-39* (SAQOL-39) [40]

The RCPM [36], a test used to measure abstract reasoning, is also regarded as a nonverbal estimate of Gf [41]. The RCPM is made up of a series of diagrams or designs with a part missing, and the participant is asked to choose the shape to complete the pattern or shape from six alternatives. The Greek version of RCPM was administered as adapted by [42]. Every correctly solved pattern was given 1 point, with a total score range between 0 and 36 [43].

The *BDAE-SF* [30] has been standardized in Greek and is culturally appropriate [44]. It includes five subtests:

1. Conversational and expository speech such as simple social responses, free conversation, and picture description
2. Auditory comprehension including word comprehension, commands, and complex ideational material
3. Oral expression, such as automatized sequences, single word repetitions, repetitions of sentences, responsive naming, the Boston Naming Test–Short Form (BNT-SF), and screening of special categories
4. Reading, including letter and number recognition, picture-word matching, basic oral word reading, oral reading of sentences with comprehension, and reading comprehension of sentences and paragraphs
5. Writing, including mechanics, dictation writing of primer words, regular phonics and common irregular forms, written naming, narrative writing mechanics, written vocabulary access, syntax, and adequacy of content

For the purposes of this study, only subtests 1-4 were administered and results were analysed in accordance with the test manual.

The *RehaCom Working Memory Screening* module is a tool used to assess both simple WM span (simple information holding) and the retention and processing of visual-spatial information. When the WM screening task is initiated, ten dots are presented in a circular arrangement. Individual dots sequentially turn red and fade. The first sequence consists of two random dots out of the ten lighting up in a particular order to be repeated correctly. When selected correctly, the number of dots increases in the next sequence. In sum, the task is to memorize the presented sequence of dots lighting up. The WM screening subtest ends after two consecutive incorrect sequence responses or after 7 minutes. The visual-spatial memory span is measured by the maximum length of the memorized dot patterns that can be reproduced immediately without errors. Additionally, the participant's memory span is calculated based on the highest sequence length measured in number of dots, reproduced without mistakes in position and order, and it is confirmed by completing two consecutive sequences with the same number of dots.

A *personal stroke narrative* was elicited by asking the participant to describe how his stroke occurred [37]. The sample was transcribed and analysed using the *Shewan Spontaneous Language Analysis* (SSLA) system [45] in accordance with the SSLA protocol. Variables for analysis included number of utterances, time (total speaking time in minutes), rate (syllables per minute), length (percentage of utterances ≤ 5 words), melody, articulation, complex sentences (percentage of utterances that contained one independent clause and one or more dependent clauses), errors (percentage of grammatical, syntactic, or morphological errors), content units (units that conveyed information), paraphasias (percentage of substitutions), repetitions, and communication efficiency (content units/time).

The *MAIN* [38] is a tool designed to evaluate narrative tell and retell skills in children but has also been used with adults with acquired language deficits associated with neurological disease for research purposes [46]. The MAIN stories consist of coloured picture sequences developed according to strict psycholinguistic criteria. While the MAIN examines narrative production at microstructure and macrostructure levels, for this study, only the macrostructure of the generated story was analysed. The primary unit for macrostructure analysis is the episode. The content of each picture sequence was designed to represent three short episodes. Each episode consists of

- a goal statement for the protagonist
- an attempt by the protagonist to reach the goal
- an outcome of the attempt in terms of the goal
- the internal states (IST) which initiate the goal and also express reactions

Each story is controlled for cognitive and linguistic complexity [38] and has a moral meaning similar to an Aesop fable. In this study, the “Baby Goats” story was used which portrayed a mother goat wanting to save her baby goat who jumped into the water but a fox jumped forward to catch the other baby goat. Then, a bird saw that the baby goat was in great danger and stopped the fox by biting its tail and chasing it away to save the baby goat. Six-coloured pictures in the form of a cartoon strip were presented, and one-episode was unfolded each time (2 pictures) for the participant to narrate a story based on the pictured stimuli. The scoring sheets of the MAIN “Baby Goats” story provided the scoring system used for the story structure components (setting, goals, attempts, outcomes, and IST). A setting statement, which gives time and place and introduces the story's protagonist, is scored with zero points for wrong or no response, 1 point for one correct response, and 2 points for reference to both time and place. This component is followed by three episodes. Each episode consists of (a) the internal states which initiate the goal and also express reactions; (b) a goal which is a statement of an idea of the protagonist to deal with the initiating event; (c) an attempt by the protagonist to reach the goal, which is an indication of action to obtain the goal; (d) an outcome of the attempt in terms of the goal, which is the event(s) following the attempt and causally linked to it; and (e) the internal states as reaction, which is a statement defining how the protagonist(s) feel or think about the outcome or an action resulting from an emotional response [38]. The story output was transcribed verbatim, and it was analysed using a scoring system of 17 points for story structure components in production, following the guidelines for assessment, and guided by the information on the provided scoring sheets.

The *Procedural Discourse* task is considered a semispontaneous speech production task that assesses discourse ability following the main concept analysis (MCA) procedure [39]. The MCA enumerates the speaker's ability to communicate the overall idea of an occasion, and it provides a way to evaluate the generated precision and completeness of the critical concepts of the shared topic. The participant was instructed to verbally provide all the required steps to be taken in order to prepare a sandwich. The generated language sample was analysed using the MCA procedure referring to the ten main concepts. The total number of main concepts expected to be produced was analysed and measured based on the concept content as listed below:

1. Get the bread out
2. Get two slices of bread//halved bread
3. Get the butter
4. Get the (rest of the ingredients, i.e., ham and cheese)
5. Get a knife
6. Put/place the bread on the plate
7. Put/spread butter on bread
8. Put the ingredients (i.e., ham and cheese) on bread
9. Put the two pieces together
10. Cut the sandwich in pieces

The first five steps comprise concepts concerning retrieving the ingredients needed, the following four steps include concepts concerning ingredient assembly, and the final concept describes the final appearance of the target (sandwich) prior to serving it. The procedure output was transcribed verbatim, and it was analysed using a binary scoring system of “1” for correct information and “0” for incorrect/missing information.

The *SAQOL-39*g has been translated and culturally adapted in Greek for use in Greece with PWA [47]. The Greek SAQOL-39g shows good reliability and validity [48] as a measure of health-related quality of life in people with stroke, including those with aphasia. An interview with the participant and the first author took place prior to the therapy study where the SAQOL-39 was used to collect the relevant information.

### 2.4. Therapy Procedure

**Figure 3:**
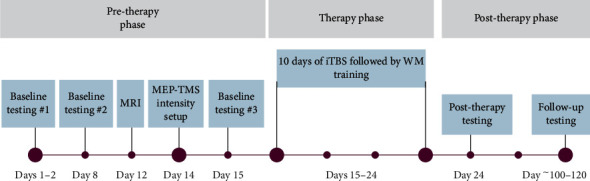
Study design overview.

The participant completed ten (10) approximately 45-minute-long treatment sessions comprising of iTBS, immediately followed by RehaCom WM training over a span of 10 consecutive days, including weekends. Within each treatment session, approximately 15 minutes were devoted for setting up the participant with the TMS equipment and iTBS application, and 30 minutes were devoted to the RehaCom WM training task. The treatment regimen is depicted in Figure 3 below.

#### 2.4.1. Pretherapy or “Baseline” Testing Phase

During the pretherapy baseline phase, the purpose was to establish the level of performance prior to treatment so that the effects of treatment on the task could be clearly measured. Seven outcome measures were used, and the information was collected three times, one week apart, prior to the therapy phase. Preceding the therapy phase, a T1-weighted MRI image was obtained of C. S.'s brain in order to accurately locate the target stimulation site using the Visor 2.0 neuronavigation system (ANT NEURO). Neuronavigated positioning of the stimulation coil allowed for repeated accuracy throughout the study.

*(1) Transcranial Magnetic Stimulation (TMS) Equipment*. Single-pulse TMS and intermittent theta-burst stimulation (iTBS) were delivered over the motor cortex and the left dorsolateral prefrontal cortex (LDLPFC), respectively, with the Magstim Rapid2® stimulator (Magstim Co., Wales, UK) connected to a 70 mm figure-8 air cooled coil. Biphasic TMS pulses were delivered with a posterior-to-anterior (P-A) current direction in both, single-pulse TMS and iTBS. The treatment intensity of TMS was individually adjusted the participant's resting motor threshold (RMT). RMT is the minimal intensity at which TMS of motor cortex produces a reliable motor evoked potential (MEP) of minimal amplitude in the target muscle. The MEP was determined with a surface electromyography (EMG) response in the ‘target' muscle, through the placement of EMG leads over the first dorsal interosseous (FDI) muscle of the left hand. Full muscle relaxation was maintained through visual and online EMG monitoring. The coil was positioned at 45-degree rotation in relation to the parasagittal plane to induce P-A current in the underlying cortex. The motor “hotspot” was determined with a TMS intensity ranging from 45% to 50% of the maximum stimulator output, whereby single-pulse stimuli were delivered at varying positions across the scalp near the primary motor cortex (M1) while guided by a neuronavigation system (ANT NEURO) using each participant's recent anatomical MRI image. The motor “hotspot” was determined as the position on the scalp that yielded two consecutive MEPs with greater amplitude than the surrounding positions. The location within the left motor cortex that consistently elicited MEPs in the relaxed right FDI muscle was then defined as the motor hotspot. The coil was then placed over the defined target to obtain a MEP in the FDI of at least 50 *μ*V in five or more of 10 consecutive stimulations of the left hand [49]. For this study, a computerized adaptive parameter estimation through sequential testing (PEST) [50] with the software TMS Motor Threshold Assessment Tool, MTAT 2.0, developed by Awiszus and Borckardt et al. [50], was used to determine the RMT. The MTAT 2.0 freeware was obtained online (http://www.clinicalresearcher.org/software.html), and the option for assessment without prior information was selected. No other parameters were changed on the software.

#### 2.4.2. Therapy Phase

*(1) Transcranial Magnetic Stimulation: iTBS Application*. The figure-8 coil was positioned tangentially to the skull, with the handle parallel to the sagittal axis pointing occipitally. The iTBS treatment consisted of bursts of three pulses at 50 Hz given every 200 milliseconds in two second trains, repeated every 10 seconds over 200 seconds for a total of 600 pulses [51]. Based on the participant's recent MRI images, the Visor 2.0 neuronavigation suite (ANT-Neuro, Enschede, Netherlands) was used for image preprocessing, tissue segmentation, and registration into standard stereotaxic space. The stimulation target was defined in the left DLPFC by using the Talairach coordinates *x* = −39, *y* = 34, and *z* = 27 [21, 52]. This technology enabled the reliable three-dimensionally precise reapplication of rTMS throughout the study. The participant received one session of iTBS each day for 10 consecutive days.

*(2) RehaCom WM Training Equipment*. Immediately following the iTBS session, the participant received 30 minutes WM training using the RehaCom Working Memory (WOME) software package (Hasomed GmbH, DE.). RehaCom WOME is a software package developed to train and improve WM performance. The WM training task involved card presentation in the form of a card game, using a complete card deck of 52 cards and consisting of different levels of difficulty. Three hierarchically ordered modules were designed to exercise the main components of WM on the basis of a card game: (a) storage systems, involving the maintenance of information; (b) selective attention, involving memorizing selective parts of information and inhibiting others; and (c) central executive/manipulation processes, involving active operating with the content retained in WM [53]. RehaCom WOME training involves the memorization and manipulation of an increasing number of visually presented playing cards on a computer screen. Throughout the early levels of training, the participant is required to memorize a short series of cards and reproduce it in the same order, while at higher levels additional tasks are introduced to influence the memory process (e.g., memorize only the cards of a certain suit from a presentation of various cards). In total, there are 70 levels of difficulty. Feedback is constantly provided by the software, and the degree of difficulty is adapted based on the participant's performance level. The sessions were implemented in a quiet room, and C.S. responded on a Lenovo touchscreen laptop.

### 2.5. Posttherapy/Follow-Up Phase

The posttherapy/follow-up phase consisted of two time points. The outcome measures were administered immediately after the completion of the last day of treatment (10^th^ day) and at 3 months posttreatment at the follow-up stage. The purpose of immediate posttesting was to determine short-term efficacy and of the follow-up to determine long-term effects. The exact date of the follow-up was dependent on the participant's availability when contacted to set-up the appointment. The same battery of tools was used as with the baseline phase:

1. The Greek BDAE-SF [30]
2. The RCPM [42]
3. The MAIN [38]
4. A Procedural Discourse task [39]
5. A personal stroke narrative [37]
6. The RehaCom Working Memory Screening Task
7. The Greek SAQOL-39 [48]

## 3. Results

The Statistical Package for Social Sciences (IBM SPSS 25) was used for all the data and exploratory analysis. Analyses of individual data were conducted using the weighted statistics (WEST) method, and descriptive results' analysis was used where a statistical analysis was not suitable. Specifically, the “WEST-Trend” and “WEST-ROC” (one tailed) procedures [54] were applied. In order to evaluate the treatment effects and the rate of change, the level of performance prior to treatment is established by taking at least two probes [54]. A linear trend in improvement may be documented using the WEST-Trend procedure, while the amount of change in the treated (short-term) versus the untreated periods (long-term) may be documented using the WEST-ROC analyses. The WEST-ROC and WEST-Trend were used to analyse the data from the Greek BDAE-SF, the RCPM, the MAIN, and the Procedural Discourse. Results from the SSLA, the RehaCom WM screening, and the SAQOL-39g assessments are reported but a statistical analysis was not performed.

### 3.1. Near-Transfer Effects of iTBS to the LDLPFC Combined with WM Training

**Figure 4:**
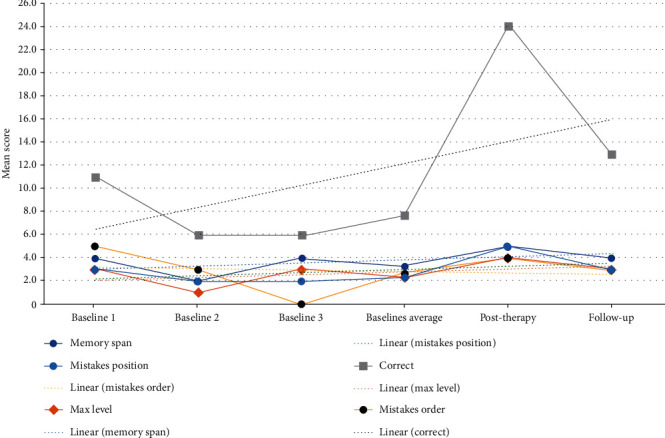
Schematic representation of C.S.'s raw scores on the *RehaCom* WM screening task.

The results of the *RehaCom WM* screening show a positive linear trend (Figure 4) on all the tasks assessed with a more prominent trend for improvement in the correct responses task. The baseline average (avg.) was compared to the posttherapy and follow-up results (Table 2).

C.S. did not show an overall significant improvement in the RCPM results. However, a statistically significant trend for improvement was shown in *Subtest AB*, (*t*(11) = 1.82, *p* = 0.048) but the WEST-ROC showed that the difference between the treated and untreated periods was nonsignificant (*t*(11) = 0.64, *p* = 0.268). Results are shown in percentage correct in Table 3.

### 3.2. Far-Transfer Effects in PWA of iTBS to the LDLPFC Combined with WM Training

To investigate whether TMS and WM training generalized to untrained receptive and expressive language and functional communication tasks, statistical analysis was performed on results from (i) the *BDAE-SF*, (ii) the *Procedural Discourse* task, and (iii) the *MAIN* telling task (Table 4). The personal stroke narrative was analysed using the SSLA.

- Statistical analysis of the *BDAE* subtests revealed a significant overall trend for improvement only for the *Boston Naming Test* (*t*(14) = 1.82, *p* = 0.045) while the difference between the treated and untreated periods was nonsignificant (*t*(14) = 0.27, *p* = 0.396)
- Statistical analysis of the participant's *BDAE Reading* subtest showed a significant trend for improvement (*t*(6) = 2.00, *p* = 0.046), but difference was nonsignificant between the treated and untreated periods (*t*(6) = 0.30, *p* = 0.389)
- Statistical analysis of the participant's *Procedural Discourse* task showed that there were no differences in the number of responses between the five periods
- There was an overall trend for improvement on the *MAIN*, but this did not reach significance (*t*(16) = 1.37, *p* = 0.095), as well as between the treated and untreated periods (*t*(16) = 1.24, *p* = 0.116). Improvement was noted for (a) the IST event as initiating of the second episode during posttherapy and follow-up, (b) the IST event as initiating of the third episode during posttherapy and follow-up, and (c) and IST as reaction of the second episode during follow-up

(v) C.S.'s stroke narrative (spontaneous language sample) was analysed using the SSLA protocol [45] which is designed to describe and quantify connected speech. The baseline average (avg) was compared with the posttesting and follow-up results. There was a 1% increase in the number of utterances produced between baseline avg and posttherapy and a 7% increase between baseline avg and follow-up. The rate of speech improved from 116.76 syllables per minute to 141.60 at posttherapy and to 152.22 at follow-up. The sentence length, which reflects the use of more than 5 words in the produced utterances, improved by 22% between baseline avg and follow-up. A 7% improvement was noted in sentence complexity between baseline avg and follow-up. Improvement was also noted between baseline avg and follow-up in the with an 11% reduction of errors. The number of content units improved from 19.33 at baseline avg to 21.00 at posttherapy and to 36.00 at follow-up. Improvement in the number of repetitions was noted with a reduction from 7% to 0% between baseline avg and posttherapy. A notable improvement in communication efficiency which reflects the rate at which information is conveyed by the speaker (number of content units divided by time), from 13.33 at baseline avg to 16.80 posttherapy and to 17.73 at follow-up. No paraphasias were produced in any of the stroke narrative samples, and the overall melody and articulation were judged to be normal (Table 5).

### 3.3. Quality of Life Effects in PWA of iTBS to the LDLPFC Combined with WM Training

With regard to investigating whether the overall QoL would improve after the treatment, the self-rated SAQOL-39 was analysed by comparing the mean scores (Table 6). The participant's responses indicated that QoL based on the overall SAQOL-39 self-rated score improved between the baseline average (*M* = 3.63) and posttherapy (*M* = 4.51) by 18%, and it was maintained at follow-up (*M* = 4.23).

## 4. Discussion

The main objective was to explore the potential domains of transfer effects after stimulating the left DLPFC combined with WM training and also to measure how this affected the quality of life of the participant. In a previous pilot study [10], we found evidence signifying the possible improvement that could specifically yield from noninvasive brain stimulation programs using iTBS combined with the *RehaCom* WM training program in PWA. Studies performed on healthy aging which are usually focused on prevention, consider the use of rTMS as a tool for cognitive enhancement of the elderly with mild cognitive impairments (MCI), aimed at reversing or compensating for the cognitive deficits [55, 56]. Evidence suggests that the effects of rTMS application may work in synergy with cognitive training to give rise to greater neurocognitive enhancement [55, 57, 58], supporting the notion that cognitive rehabilitation with rTMS can be beneficial as an add-on instrument in cognitive training programs of a variety of neurological and cognitive disorders [59]. A recent review investigating the effects of rTMS in people with Alzheimer's disease and related dementias (ADRD) reported 8 new studies between the years 2016 and 2018, of which 4 of them used cognitive training as well [60]. These studies reported significant improvements in global cognition and memory when measured with the following neuropsychological tests: Alzheimer's Disease Assessment Scale-cognitive (ADAS-Cog), Mini Mental State Exam (MMSE), Addenbrooke Cognitive Examination (ACE), Apathy Evaluation Scale (AES-C), Blessed Dementia Scale (BDS), Clinical Global Impression (CGI), Clinical Global Impression of Change (CGIC), Digit Symbol Substitution Test (DSST), Montreal Cognitive Assessment (MOCA), Neuropsychiatric Inventory (NPI), Rey Auditory Verbal Learning Test (RAVLT), Trail Making Test (TMT), and Zarit Burden Scale (ZBS). Even though results suggested a potential for improvement in cognitive measures after rTMS treatments, results were mixed as to whether rTMS was significantly more effective than sham. It is believed that the inconsistency of treatment protocols and outcome measures hinders the replication of promising studies, and therefore, evidences continue to be insufficient to support the adoption of a noninvasive brain stimulation protocol to improve cognitive impairments.

In line with previous studies, findings from this investigation (Table 7) lend support to the evidence that (i) WM interacts with language abilities and deficits in WM influence language performance [10, 61], (ii) applying iTBS to the LDLPFC results in improved WM performance [10, 62, 63], (iii) computerized WM training can have positive outcomes on WM tasks [64], and (iv) aphasia has a negative effect on QoL [65].

### 4.1. Near-Transfer Effects of iTBS and WM Training

WM has been proven to be a useful indicator of cognitive-linguistic competence [66], while WM impairments have a negative impact on cognition and communication [67, 68]. It is important to highlight the fact that WM interventions have a positive impact on WM capacity, as well as on related cognitive-linguistic abilities and on cognitive-communicative deficiencies due to aging or neurological disorders [25, 69]. Furthermore, studies have demonstrated transfer of WM training to other assessments of cognition, including measures of fluid intelligence [27]. This study has revealed a trend for improvement in both WM tasks and Gf transfer, with a statistical significance of Gf as measured with the RCPM after the 10-day iTBS application to the left DLPFC followed by the 30-minute WM training. It was also hypothesised that stimulation of the DLPFC combined with WM training would result in positive “near-transfer” cognitive effects with subsequent improved scores on untreated cognitive areas (i.e., Gf). A statistically significant overall trend for improvement was found in *Subtest AB* of the *RCPM*, a nontrained measure that indicates Gf (nonverbal intelligence) improvement (*t* (11) = 1.82, *p* = 0.048), while the overall score resulted in positive nonsignificant treatment effects. These findings are consistent with research showing that significant improvements in Gf resulting from cognitive intervention combined with different transcranial electrical brain stimulation protocols [70]. Our findings support the notion that Gf can be improved with DLPFC stimulation [70] and WM training [10, 71–73]. Considering the fact that a combination of treatments was used and it is still controversial whether WM training leads to Gf improvement [74], the findings are inconclusive as to whether improvement was due to the treatment combination or to the DLPFC stimulation. It is worth investigating further whether this association is significant in future research.

### 4.2. Far-Transfer Effects of iTBS and WM Training

In the past, other groups of researchers focused on investigating improvements in *Auditory Comprehension* where they showed that WM training was used to improve receptive language abilities in PWA [75–79]. These aforementioned studies reported language improvements in tasks such as commands and naming when measured on language tests such as the Western Aphasia Battery (WAB), the Test for the Reception of Grammar (TROG), and the Token Test (TT). The participant of this study had been experiencing mild expressive aphasia with STM difficulties. Results of the treatment showed a significant overall trend for improvement in the *Boston Naming Test* of the *BDAE Oral Expression* subtest. These results tie in well with previous studies where noninvasive brain stimulation to the left prefrontal cortex generated verbal working memory improvements and naming facilitation [10, 80]. Additionally, there was a significant trend for improvement in the overall *BDAE Reading* subtest; although when the individual tasks were analysed, significance was not reached. These results are in agreement with our previous research in which reading abilities were improved [10]. To the best of our knowledge, no other studies so far have investigated language improvements following iTBS combined with WM training with regard to naming or reading.

Two types of tasks were used to collect narrative discourse: the Baby Goat story from the *MAIN* [38] and a personal stroke narrative [37]. The participant showed a nonsignificant trend for improvement in the narrative, specifically showing improvement in the IST initiating structure of the story. Evidence supports that WM impairment in PWA adversely affects their ability to produce macrolinguistic narrative components [81] and higher scores on WM measures are associated with better discourse production abilities in people with brain injury [82]. The SSLA system (Shewan, 1988) was used in this study to examine the broad spectrum of language variables, in order to analyse and quantify the personal stroke narrative. The participant showed a positive linear trend in the *Rate* of speech and *Sentence Complexity*, while there was a negative linear trend in *Errors* indicating improvement. Although linguistic analysis was not generally used in the aphasia treatment literature to evaluate changes in linguistic complexity, there is an increase in research on the topic over the last few years [83]. Even though language sample analysis is commonly used to evaluate linguistic development in children [84], verbal abilities have been examined by analysing language samples [85, 86]. Few studies in the aging literature involving language analysis by obtaining oral language samples through prompts or through conversation [87]. A positive trend towards improvement in discourse was noted for both language tasks, which are consistent with the results of our previous pilot study [10].

Procedural Discourse analysis was based on the analysis developed by Richardson and Dalton [39]. The participant did not show any changes in the responses across the five periods in the *Procedural Discourse* task. Although improvements in these tasks did not reach significance, findings are in agreement with research from the aphasia literature on discourse tasks [88–91]. From the aforementioned studies, only one study was specifically directed to procedural discourse [92], with the more recent studies [88, 89, 91] exploring all aspects of discourse production, including narratives, revealing that as aphasia severity increases, quality and quantity of relevant discourse decrease. The reduction in sentence complexity experienced by PWA has also been shown to differ at a single word and semantic level, which is likely to affect procedural discourse, suggesting that PWA communicate less information in language in a context where spoken language may already be structurally less complex [93]. PWA use fewer correct information units (CIU; i.e., any single word, intelligible, informative, and relevant in context) in discourse than neurologically healthy people (NHP) [94], as well as fewer types and tokens of spatial language in spatial tasks than NHP [95].

The investigation of the use of iTBS in aphasia rehabilitation poststroke continues to be very limited. There are a few studies though that provide evidence for its efficacy. When iTBS was applied in eight individuals with chronic aphasia poststroke for five consecutive days over the course of two weeks, six patients showed significant pre-/post-rTMS improvements in semantic fluency in which the participants were able to generate more appropriate words when prompted with a semantic category. Additionally, increases in the left frontotemporoparietal language networks with a significant left hemispheric shift in the left frontal, left temporoparietal, and global language regions were reported at the pre-/post-rTMS fMRI maps of the study [96]. Further to iTBS investigations, Georgiou et al. [97] recently reported promising findings of neuronavigated continuous theta-burst stimulation (cTBS) over the right pars triangularis (Tr) as a standalone treatment for two individuals with chronic poststroke aphasia in which cTBS was carried over 10 consecutive days for 40 secs per sessions. Their results revealed improvement in language skills in the posttreatment phase, which reverted to baseline scores at follow-up and improvement in the QoL [97].

The self-reported SAQOL-39 questionnaire was administered to C.S. in an interview format to rate his current levels of QoL. A positive linear trend for improvement in the overall QoL across time was noted, which was also maintained 3 months after the treatment, with prominent improvements in the communication and psychosocial fields. This is in line with the QoL literature that the improvement in the severity of language deficits has a positive effect in the QoL [98]. Moreover, the results are consistent with what has been found in previous research that nonverbal cognitive impairments may significantly affect QoL in PWA and are potentially important predictors to improvement [99].

## 5. Conclusions

The relationship between treatment of WM deficits and the impact on language abilities in poststroke aphasia was investigated. The purpose was to determine whether WM is improved after applying excitatory noninvasive brain stimulation (iTBS) followed by computerized WM training. Furthermore, it was important to decipher whether WM improvements lead to near-transfer on unpractised WM tasks and nonverbal intelligence and far-transfer effects on language tasks, narratives, functional communication and QoL. Overall, we report improvement in memory, fluid intelligence, language, and QoL after 10 sessions of iTBS combined with computerized WM training in a single case study with no adverse effects during treatment and at follow-up periods. As it is widely acknowledged amongst rehabilitation professionals, the deficits acquired after a stroke persist for long periods and positive effects are accomplished at a slower rate. The results of this study are indicative that computerized WM training and stimulation of the LDLPFC are areas that have a positive effect in neurorehabilitation of PWA after a stroke. Improvements were noted in only 10 days and even though not all the benefits were maintained at follow-up (3 months post), the positive linear trendlines signify that there is efficacious treatment potential, which requires further exploration towards facilitating language recovery in PWA. It is important to consider that aphasia treatment programs could benefit from neurorehabilitation to increase the pace of recovery, especially during the first months of rehabilitation. The results of this study provide a preliminary indication that stimulation of the LDLPFC combined with computerized WM training after left hemisphere stroke may generalize to language improvements.

## Figures and Tables

**Table 1:** Participant inclusion and exclusion criteria.

Inclusion criteria	Exclusion criteria
(a) Native speaker of Cypriot-Greek	(a) Severe aphasia diagnosed using the Greek version of the Boston Diagnostic Aphasia Examination – Short Form (BDAE-SF) [30]
(b) Age 21-79 y.o	(b) Damaged dorsolateral prefrontal cortex area as identified in the MRI
(c) First-time single left hemisphere stroke	(c) Traumatic brain injury
(d) Presence of aphasia	(d) History of psychiatric or other neurological illness
(e) Right-hand dominance	(e) Depression
(g) Adequate single-word comprehension	(f) Epilepsy/seizures
	(g) Pregnancy
	(h) Colour-blindness or other visual disorders/visual neglect
	(i) Hearing loss
	(k) Significant general medical problems including liver, cardiac, or renal dysfunctions (l) Present or past alcohol or drug abuse (j) Metal or medical implants (i.e., cardiac pacemakers)

**Table 2:** *RehaCom* WM screening raw scores by the subcategory and study phase.

	Baseline 1	Baseline 2	Baseline 3	Baseline average	Posttherapy	Follow-up
Memory span	4	2	4	3	5	4
Max level	3	1	3	2	4	3
Correct	11	6	6	8	24	13
Mistakes order	5	3	0	3	4	3
Mistakes position	3	2	2	2	5	3

**Table 3:** Raw scores (% correct) on the nonverbal intelligence outcomes at posttreatment and follow-up compared to the baseline for C.S.

	Baseline 1	Baseline 2	Baseline 3	Posttherapy	Follow-up
Mean RCPM	75.00%	69.44%	72.22%	77.78%	77.78%
Subtest A	83.33%	75.00%	75.00%	83.33%	75.00%
Subtest AB	83.33%	83.33%	83.33%	91.67%	100.00%
Subtest B	58.33%	50.00%	58.33%	58.33%	58.33%

**Table 4:** Raw scores (% correct) on the language outcomes at posttreatment and follow-up compared to baseline for C.S.

	Baseline 1	Baseline 2	Baseline 3	Posttherapy	Follow-up
Boston naming test	66.67%	73.33%	73.33%	80.00%	86.67%
BDAE reading subtest	86.94%	90.28%	90.28%	90.28%	94.44%
Language discourse—MAIN	47.06%	47.06%	47.06%	58.82%	64.71%

**Table 5:** Raw scores for personal stroke narrative analysis based on the SSLA.

	Baseline 1	Baseline 2	Baseline 3	Baseline avg	Post-therapy	Follow-up
Utterances	12.00	14.00	7.00	11.00	12.00	18.00
Rate	99.37	152.41	98.50	116.76	141.60	152.22
Length	58%	43%	14%	39%	50%	17%
Melody	3.00	3.00	3.00	3.00	2.00	4.00
Articulation	7.00	7.00	7.00	7.00	7.00	7.00
Complexity	42%	50.00%	72%	54%	33%	61%
Errors	17%	57%	57%	44%	50%	33%
C.U.s	19.00	22.00	17.00	19.33	21.00	36.00
Paraphasias	0%	0%	0%	0%	0%	0%
Repetitions	0%	21%	0%	7%	0%	22%
Communication efficiency	12.03	15.17	12.78	13.33	16.80	17.73

**Table 6:** Quality of life for C.S. at pretreatment (baseline) at posttreatment and 3-month follow-up using the SAQOL-39g.

Item (max score: 5)	Baseline 1	Baseline 2	Baseline 3	Post-therapy	Follow-up
SAQOL-39 g mean	3.44	3.41	4.05	4.51	4.23
Physical	4.31	3.88	4.56	4.75	4.88
Communication	3.57	3.71	4.14	4.43	4.14
Psychosocial	2.50	2.88	3.56	4.38	3.56

**Table 7:** Task performance after the treatment.

Task	Treatment results
WM–number of correct responses	Improved and maintained
RCPM	Non-significant improvement and maintenance in subtest AB
BDAE auditory comprehension	No improvement
BDAE oral expression	Significant improvement and maintenance in the Boston naming test
BDAE reading	Nonsignificant improvement and maintenance in matching cases/scripts and word identification Significant trend for improvement in the overall reading task
Narrative (MAIN)	Non-significant improvement and maintenance
Procedural discourse	No improvement
Communication efficiency (SSLA)	Improvement and maintenance
QoL	Improvement and maintenance

## Data Availability

The data used to support the findings of this study are available from the corresponding author upon request.

## References

[1] Barker A. T., Jalinous R., Freeston I. L. (1985). Non-invasive magnetic stimulation of human motor cortex. *The Lancet*.

[2] Bagherzadeh Y., Khorrami A., Zarrindast M. R., Shariat S. V., Pantazis D. (2016). Repetitive transcranial magnetic stimulation of the dorsolateral prefrontal cortex enhances working memory. *Experimental Brain Research*.

[3] Baddeley A. (2003). Working memory: looking back and looking forward. *Nature Reviews. Neuroscience*.

[4] Dehn M. J. (2017). How working memory enables fluid reasoning. *Applied Neuropsychology: Child*.

[5] Hedden T., Yoon C. (2006). Individual differences in executive processing predict susceptibility to interference in verbal working memory. *Neuropsychology*.

[6] Fukuda K., Vogel E., Mayr U., Awh E. (2010). Quantity, not quality: the relationship between fluid intelligence and working memory capacity. *Psychonomic Bulletin & Review*.

[7] Johnson M. K., McMahon R. P., Robinson B. M. (2013). The relationship between working memory capacity and broad measures of cognitive ability in healthy adults and people with schizophrenia. *Neuropsychology*.

[8] Kane M. J., Engle R. W. (2002). The role of prefrontal cortex in working-memory capacity, executive attention, and general fluid intelligence: an individual-differences perspective. *Psychonomic Bulletin & Review*.

[9] Lee M. S., Kim B. S. (2021). Effects of working memory intervention on language production by individuals with dementia. *Neuropsychological Rehabilitation*.

[10] Kranou-Economidou D., Kambanaros M. (2020). Combining intermittent theta burst stimulation (iTBS) with computerized working memory training to improve language abilities in chronic aphasia: a pilot case study. *Aphasiology*.

[11] Kasselimis D. S., Neurology Department, Eginition Hospital, National and Kapodistrian University of Athens, Greece (2015). Working memory and aphasia. *International Journal of Neurology Research*.

[12] Kim C., Kroger J. K., Calhoun V. D., Clark V. P. (2015). The role of the frontopolar cortex in manipulation of integrated information in working memory. *Neuroscience Letters*.

[13] Osaka N., Osaka M., Kondo H., Morishita M., Fukuyama H., Shibasaki H. (2004). The neural basis of executive function in working memory: an fMRI study based on individual differences. *NeuroImage*.

[14] Owen A. M., McMillan K. M., Laird A. R., Bullmore E. (2005). N-back working memory paradigm: a meta-analysis of normative functional neuroimaging studies. *Human Brain Mapping*.

[15] Chein J. M., Moore A. B., Conway A. R. A. (2011). Domain-general mechanisms of complex working memory span. *NeuroImage*.

[16] Chai W. J., Abd Hamid A. I., Abdullah J. M. (2018). Working memory from the psychological and neurosciences perspectives: a review. *Frontiers in Psychology*.

[17] Baddeley A. D., Allen R. J., Hitch G. J. (2011). Binding in visual working memory: the role of the episodic buffer. *Neuropsychologia*.

[18] Courtney S. M. (2004). Attention and cognitive control as Emergent properties of information representation in working memory. *Cognitive, Affective, & Behavioral Neuroscience*.

[19] Pessoa L., Gutierrez E., Bandettini P., Ungerleider L. (2002). Neural correlates of visual working memory: fMRI amplitude predicts task performance. *Neuron*.

[20] Owen A. M., Evans A. C., Petrides M. (1996). Evidence for a two-stage model of spatial working memory processing within the lateral frontal cortex: a positron emission tomography study. *Cerebral Cortex*.

[21] Wager T. D., Smith E. E. (2003). Neuroimaging studies of working memory:. *Cognitive, Affective, & Behavioral Neuroscience*.

[22] Cho S. S., Strafella A. P. (2009). rTMS of the left dorsolateral prefrontal cortex modulates dopamine release in the ipsilateral anterior cingulate cortex and orbitofrontal cortex. *PLoS One*.

[23] D’Esposito M., Postle B. R. (2015). The cognitive neuroscience of working memory. *Annual Review of Psychology*.

[24] Au J., Sheehan E., Tsai N., Duncan G. J., Buschkuehl M., Jaeggi S. M. (2015). Improving fluid intelligence with training on working memory: a meta-analysis. *Psychonomic Bulletin & Review*.

[25] Klingberg T. (2010). Training and plasticity of working memory. *Trends in Cognitive Sciences*.

[26] Zhao X., Wang Y., Liu D., Zhou R. (2011). Effect of updating training on fluid intelligence in children. *Chinese Science Bulletin*.

[27] Jaeggi S. M., Buschkuehl M., Jonides J., Perrig W. J. (2008). Improving fluid intelligence with training on working memory. *Proceedings of the National Academy of Sciences*.

[28] Cattell R. B. (1971). *Abilities: Their Structure, Growth, and Action*.

[29] Gray J. R., Chabris C. F., Braver T. S. (2003). Neural mechanisms of general fluid intelligence. *Nature Neuroscience*.

[30] Messinis L., Panagea E., Papathanasopoulos P., Kastellakis A. (2013). *Boston diagnostic aphasia examination-short form in Greek language*.

[31] Keel J. C., Smith M. J., Wassermann E. M. (2001). A safety screening questionnaire for transcranial magnetic stimulation. *Clinical Neurophysiology*.

[32] Albert M. L. (1973). A simple test of visual neglect. *Neurology*.

[33] Oldfield R. C. (1971). The assessment and analysis of handedness: The Edinburgh inventory. *Neuropsychologia*.

[34] Beck A. T., Ward C. H., Mendelson M., Mock J., Erbaugh J. (1961). An inventory for measuring depression. *Archives of General Psychiatry*.

[35] Giannakou M., Roussi P., Kosmides M. E., Kiosseoglou G., Adamopoulou A., Garyfallos G. (2013). Adaptation of the beck depression inventory-II to Greek population. *Hellenic Journal of Psychology*.

[36] Raven J. (2000). The Raven’s progressive matrices: change and stability over culture and time. *Cognitive Psychology*.

[37] Kambanaros M. (2019). Evaluating personal stroke narratives from bilingual Greek-English immigrants with aphasia. *Folia Phoniatrica et Logopaedica*.

[38] Gagarina N. V., Klop D., Kunnari S. (2012). Multilingual Assessment Instrument for Narratives. *ZAS papers in linguistics*.

[39] Richardson J. D., Dalton S. G. (2016). Main concepts for three different discourse tasks in a large non-clinical sample. *Aphasiology*.

[40] Hilari K., Lamping D. L., Smith S. C., Northcott S., Lamb A., Marshall J. (2009). Psychometric properties of the Stroke and Aphasia Quality of Life Scale (SAQOL-39) in a generic stroke population. *Clinical Rehabilitation*.

[41] Bilker W. B., Hansen J. A., Brensinger C. M., Richard J., Gur R. E., Gur R. C. (2012). Development of abbreviated nine-item forms of the Raven’s standard progressive matrices test. *Assessment*.

[42] Sideridis G., Antoniou F., Mouzaki A., Simos P. (2015). Raven’s educational CPM/CVS. *Athens Motiv.*.

[43] Papantoniou G., Moraitou D., Dinou M., Katsadima E., Savvidou E., Foutsitzi E. (2016). Comparing the latent structure of the children’s category test-level 1 among young children and older adults: a preliminary study. *Psychology*.

[44] Tsapkini K., Vlahou C. H., Potagas C. (2010). Adaptation and Validation of Standardized Aphasia Tests in Different Languages: Lessons from the Boston Diagnostic Aphasia Examination–Short Form in Greek. *Behavioural Neurology*.

[45] Shewan C. M. (1988). The _Shewan Spontaneous Language Analysis_ (SSLA) system for aphasic adults: Description, reliability, and validity. *Journal of Communication Disorders*.

[46] Karpathiou N., Papatriantafyllou J., Kambanaros M. (2018). Bilingualism in a case of the non-fluent/agrammatic variant of primary progressive aphasia. *Frontiers in Communication*.

[47] Kartsona A., Hilari K. (2007). Quality of life in aphasia: Greek adaptation of the stroke and aphasia quality of life scale - 39 item (SAQOL-39). *Europa Medicophysica*.

[48] Efstratiadou E. A., Chelas E. N., Ignatiou M., Christaki V., Papathanasiou I., Hilari K. (2012). Quality of life after stroke: evaluation of the Greek SAQOL-39g. *Folia Phoniatrica et Logopaedica*.

[49] Rossini P. M., Burke D., Chen R. (2015). Non-invasive electrical and magnetic stimulation of the brain, spinal cord, roots and peripheral nerves: Basic principles and procedures for routine clinical and research application. An updated report from an I.F.C.N. Committee. *Clinical Neurophysiology*.

[50] Borckardt J. J., Nahas Z., Koola J., George M. S. (2006). Estimating resting motor thresholds in transcranial magnetic stimulation research and practice: a computer simulation evaluation of Best Methods. *The journal of ECT*.

[51] Huang Y. Z., Edwards M. J., Rounis E., Bhatia K. P., Rothwell J. C. (2005). Theta burst stimulation of the human motor cortex. *Neuron*.

[52] Barbey A. K., Koenigs M., Grafman J. (2013). Dorsolateral prefrontal contributions to human working memory. *Cortex*.

[53] Weicker J., Hudl N., Frisch S. (2018). WOME: theory-based working memory training - a placebo-controlled, double-blind evaluation in older adults. *Frontiers in Aging Neuroscience*.

[54] Howard D., Best W., Nickels L. (2015). Optimising the design of intervention studies: critiques and ways forward. *Aphasiology*.

[55] Clark V. P., Parasuraman R. (2014). Neuroenhancement: enhancing brain and mind in health and in disease. *NeuroImage*.

[56] Cotelli M., Manenti R., Zanetti O., Miniussi C. (2012). Non-pharmacological intervention for memory decline. *Frontiers in human neuroscience*.

[57] McKendrick R., Ayaz H., Olmstead R., Parasuraman R. (2014). Enhancing dual-task performance with verbal and spatial working memory training: continuous monitoring of cerebral hemodynamics with NIRS. *NeuroImage*.

[58] Bentwich J., Dobronevsky E., Aichenbaum S. (2011). Beneficial effect of repetitive transcranial magnetic stimulation combined with cognitive training for the treatment of Alzheimer’s disease: a proof of concept study. *Journal of Neural Transmission*.

[59] Vallar G., Bolognini N. (2011). Behavioural facilitation following brain stimulation: implications for neurorehabilitation. *Neuropsychological Rehabilitation*.

[60] Buss S. S., Fried P. J., Pascual-Leone A. (2019). Therapeutic noninvasive brain stimulation in Alzheimer’s disease and related dementias. *Current Opinion in Neurology*.

[61] Murray L. L. (2012). Direct and indirect treatment approaches for addressing short-term or working memory deficits in aphasia. *Aphasiology*.

[62] Hoy K. E., Bailey N., Michael M. (2016). Enhancement of working memory and task-related oscillatory activity following intermittent theta burst stimulation in healthy controls. *Cerebral Cortex*.

[63] Demeter E., Mirdamadi J. L., Meehan S. K., Taylor S. F. (2016). Short theta burst stimulation to left frontal cortex prior to encoding enhances subsequent recognition memory. *Cognitive, Affective, & Behavioral Neuroscience*.

[64] Lundqvist A., Grundstrm K., Samuelsson K., Rönnberg J. (2010). Computerized training of working memory in a group of patients suffering from acquired brain injury. *Brain Injury*.

[65] Manning M., MacFarlane A., Hickey A., Franklin S. (2019). Perspectives of people with aphasia post-stroke towards personal recovery and living successfully: a systematic review and thematic synthesis. *PLoS One*.

[66] Oberauer K., Süß H. M., Wilhelm O., Wittmann W. W. (2008). Which working memory functions predict intelligence?. *Intelligence*.

[67] Alloway T. P. (2009). Working memory, but not IQ, predicts subsequent learning in children with learning difficulties. *European Journal of Psychological Assessment*.

[68] von Bastian C. C., Oberauer K. (2014). Effects and mechanisms of working memory training: a review. *Psychological Research*.

[69] Morrison A. B., Chein J. M. (2011). Does working memory training work? The promise and challenges of enhancing cognition by training working memory. *Psychonomic Bulletin & Review*.

[70] Brem A. K., Almquist J. N. F., Mansfield K. (2018). Modulating fluid intelligence performance through combined cognitive training and brain stimulation. *Neuropsychologia*.

[71] Engle R. W., Laughlin J. E., Tuholski S. W., Conway A. R. A. (1999). Working memory, short-term memory, and general fluid intelligence: a latent-variable approach. *Journal of Experimental Psychology. General*.

[72] Friedman N. P., Miyake A., Corley R. P., Young S. E., DeFries J. C., Hewitt J. K. (2006). Not all executive functions are related to intelligence. *Psychological Science*.

[73] Unsworth N., Fukuda K., Awh E., Vogel E. K. (2014). Working memory and fluid intelligence: capacity, attention control, and secondary memory retrieval. *Cognitive Psychology*.

[74] Harrison T. L., Shipstead Z., Hicks K. L., Hambrick D. Z., Redick T. S., Engle R. W. (2013). Working memory training may increase working memory capacity but not fluid intelligence. *Psychological Science*.

[75] Eom B., Sung J. E. (2016). The effects of sentence repetition–based working memory treatment on sentence comprehension abilities in individuals with aphasia. *American Journal of Speech-Language Pathology*.

[76] Harris L., Olson A., Humphreys G. (2014). The link between STM and sentence comprehension: a neuropsychological rehabilitation study. *Neuropsychological Rehabilitation*.

[77] Salis C. (2012). Short-term memory treatment: patterns of learning and generalisation to sentence comprehension in a person with aphasia. *Neuropsychological Rehabilitation*.

[78] Salis C., Hwang F., Howard D., Lallini N. (2017). Short-term and working memory treatments for improving sentence comprehension in aphasia: a review and a replication study. *Seminars in Speech and Language*.

[79] Zakariás L., Keresztes A., Marton K., Wartenburger I. (2018). Positive effects of a computerised working memory and executive function training on sentence comprehension in aphasia. *Neuropsychological Rehabilitation*.

[80] Jeon S. Y., Han S. J. (2012). Improvement of the working memory and naming by transcranial direct current stimulation. *Annals of Rehabilitation Medicine*.

[81] Cahana-Amitay D., Jenkins T. (2018). Working memory and discourse production in people with aphasia. *Journal of Neurolinguistics*.

[82] Youse K. M., Coelho C. A. (2005). Working memory and discourse production abilities following closed-head injury. *Brain Injury*.

[83] Bryant L., Ferguson A., Spencer E. (2016). Linguistic analysis of discourse in aphasia: a review of the literature. *Clinical Linguistics & Phonetics*.

[84] Heilmann J., Miller J. F., Nockerts A., Dunaway C. (2010). Properties of the narrative scoring scheme using narrative retells in young school-age children. *American Journal of Speech-Language Pathology*.

[85] Capilouto G., Wright H. H., Wagovich S. A. (2005). CIU and main event analyses of the structured discourse of older and younger adults. *Journal of Communication Disorders*.

[86] Shewan C. M., Henderson V. L. (1988). Analysis of spontaneous language in the older normal population. *Journal of Communication Disorders*.

[87] Kemper S., Sumner A. (2001). The structure of verbal abilities in young and older adults. *Psychology and Aging*.

[88] Andreetta S., Cantagallo A., Marini A. (2012). Narrative discourse in anomic aphasia. *Neuropsychologia*.

[89] Capilouto G. J., Wright H. H., Wagovich S. A. (2006). Reliability of main event measurement in the discourse of individuals with aphasia. *Aphasiology*.

[90] Ulatowska H., Freedman-Stern R., Doyel A., Macaluso-Haynes S., North A. (1983). Production of narrative discourse in aphasia. *Brain and Language*.

[91] Fergadiotis G., Wright H. H. (2011). Lexical diversity for adults with and without aphasia across discourse elicitation tasks. *Aphasiology*.

[92] Ulatowska H. K., North A. J., Macaluso-Haynes S. (1981). Production of narrative and procedural discourse in aphasia. *Brain and Language*.

[93] Pritchard M., Dipper L., Morgan G., Cocks N. (2015). Language and iconic gesture use in procedural discourse by speakers with aphasia. *Aphasiology*.

[94] Nicholas L. E., Brookshire R. H. (1993). A system for quantifying the informativeness and efficiency of the connected speech of adults with aphasia. *Journal of Speech and Hearing Research*.

[95] Johnson S., Cocks N., Dipper L. (2013). Use of spatial communication in aphasia. *International Journal of Language & Communication Disorders*.

[96] Szaflarski J. P., Vannest J., Wu S. W., Difrancesco M. W., Banks C., Gilbert D. L. (2011). Excitatory repetitive transcranial magnetic stimulation induces improvements in chronic post-stroke aphasia. *Medical Science Monitor*.

[97] Georgiou A., Konstantinou N., Phinikettos I., Kambanaros M. (2019). Neuronavigated theta burst stimulation for chronic aphasia: two exploratory case studies. *Clinical Linguistics & Phonetics*.

[98] Spaccavento S., Craca A., del Prete M. (2013). Quality of life measurement and outcome in aphasia. *Neuropsychiatric Disease and Treatment*.

[99] Nicholas M., Hunsaker E., Guarino A. J. (2017). The relation between language, non-verbal cognition and quality of life in people with aphasia. *Aphasiology*.

